# The routine use of nasopharyngeal airway in the setting of monitored anesthesia care during gastrointestinal endoscopy: A multi-center single blinded randomized controlled trial

**DOI:** 10.1371/journal.pone.0349946

**Published:** 2026-06-03

**Authors:** Christian Raphael, Nancy Abou Nafeh, Marc Moukarzel, Thuraya HajAli, Eliane Alhalabi, Aliyya Dabbous, Raji Naamani, Patrick Maroun, Yasser Sheib, Marie T. Aouad, Rony Al Nawwar

**Affiliations:** 1 Department of Anesthesiology, American University of Beirut Medical Center, Beirut, Lebanon; 2 Department of Internal Medicine, Division of Gastroenterology, American University of Beirut Medical Center, Beirut, Lebanon; 3 Department of Anesthesiology, Lebanese American University Medical Center-Rizk Hospital, Beirut, Lebanon; Banaras Hindu University, INDIA

## Abstract

**Background and Aims:**

Monitored anesthesia care (MAC) is commonly used for gastrointestinal (GI) endoscopy, allowing patients to breathe spontaneously while sedated. Despite its benefits, MAC is associated with respiratory adverse events, such as desaturation or the need for interventions. Limited studies have examined the effectiveness of nasopharyngeal airways (NPA) in reducing airway maneuvers and improving respiratory stability during MAC. The study evaluates the efficacy and safety of NPA in reducing the occurrence of at least one airway intervention/desaturation in patients undergoing GI endoscopy under MAC.

**Methods:**

This multi-center, single-blinded randomized controlled trial involved patients undergoing GI endoscopy under MAC. A total of 329 patients were randomly assigned to either the NPA or control group. Primary outcomes included a composite primary outcome: occurrence of at least one airway intervention/desaturation. Secondary outcomes included adverse events, satisfaction scores of patients and healthcare providers, and the joint effects of NPA use and OSA risk on the occurrence of at least one airway intervention or desaturation.

**Results:**

The NPA group showed significantly lower incidence of occurrence of at least one airway intervention/desaturation compared to controls (18.5% vs. 40.1%, *P* < 0.001). NPA significantly reduced the occurrence of at least one airway intervention/desaturation in both low and intermediate/high risk OSA groups. The reduction was stronger in low OSA risk group (OR=0.23, 95%CI [0.11–0.49]) versus intermediate/high OSA risk group (OR=0.45, 95%CI [0.21–0.95]. Health care provider satisfaction was significantly higher in the NPA group (p < 0.001). Mild, self-resolving epistaxis occurred in 3.1% of NPA patients.

**Conclusions:**

NPA use reduces airway interventions and enhances satisfaction among anesthesiologists and gastroenterologists during GI endoscopy under MAC. Routine utilization of NPAs in this context may be considered.

## Introduction

Monitored anesthesia care (MAC) is currently the dominant method of endoscopic sedation for gastroenterologists in the United States and continues to grow largely [1]. Three factors are behind the success and popularity of MAC: increased patient turnover and throughput, improved patient satisfaction, and, in some instances, reduced operating expenses [2].

Propofol is the most frequently used intravenous anesthetic. It produces varying levels of sedation along the continuum from sedation to general anesthesia [3]. The favorable pharmacokinetic properties of propofol are behind the growing popularity of MAC and improved patient satisfaction and operational efficiency [4]. The drawbacks of propofol are airway obstruction and respiratory depression, which can in turn lead to desaturation and the need for airway maneuvers to keep a stable respiratory status.

A nasopharyngeal airway (NPA) is a simple device that can be conveniently inserted into the supraglottic airway to secure an open passage [5]. Unlike an oropharyngeal airway, which is used only in unconscious patients, an NPA may be used for patients who are semiconscious (with intact cough and gag reflexes) or unconscious. It is designed to be inserted into the nasal passageway and aims to bypass upper airway obstruction at the level of the nose, nasopharynx or base of the tongue [6]. With only little reported side effects, NPA could potentially be a safe and effective way to address the unstable respiratory status that patients face. This hypothesis has been tested, and there are a few articles in the literature that study the safety and efficacy of NPA in patients undergoing GI endoscopy. A prospective study on patients with obesity undergoing gastroscopy showed that the NPA is a safe device that reduces episodes of desaturation and need for emergent treatment [7]. Another study published by Muller et. Al in 2014 that looked at a general population of patients undergoing gastroscopy showed similar positive outcomes while only studying the frequency of desaturation [1]. However, none of the published studies investigated the effect of NPA on the need for airway interventions in the general population of patients undergoing GI endoscopy.

Our proposed study is designed to evaluate the efficacy, safety, potential adverse events, and satisfaction of the gastroenterologist, anesthesiologist, and patient with the use of NPA in patients undergoing GI endoscopy procedures under MAC. We hypothesize that the use of NPA will reduce the need for airway interventions and/or desaturation during MAC with minimal associated adverse events.

## Methods

This is a prospective, multi-center, single-blinded randomized controlled trial (RCT) conducted on patients admitted for GI endoscopy (gastroscopy, colonoscopy, endoscopic ultrasound (EUS)) under MAC from November 11, 2019 till November 8, 2023 at AUBMC, and from June 1, 2022 till November 8, 2023 at LAUMC-RH.

The study received approval from the institutional review boards (center 1 protocol# BIO-2018–0549; center 2 protocol# LAUMCRH.RN1.4/Apr/2022), and written informed consent was obtained from all subjects participating in the trial. The trial was registered before patient enrollment at clinicaltrials.gov (NCT04123821, principal investigator: Christian Raphael; date of registration: October 11, 2019), and performed in accordance with the ethical standards that were set in the 1964 Declaration of Helsinki and its later amendments. This manuscript adheres to the applicable CONSORT guidelines [8].

A standard preoperative anesthesia evaluation was conducted on all patients including medical history and physical examination as per our institutional protocol. Subsequently, written informed consent was obtained from patients who met the inclusion criteria before their enrollment in the study. Informed consent was obtained in a private setting according to our institutional guidelines. The consent process included a thorough explanation of the study procedure, potential risks, and the patients’ right to withdraw from the study at any time without affecting their clinical care.

On the day of their arrival, patients were evaluated according to the inclusion/exclusion criteria. If eligible, they were approached by a member of the research team to participate in the research study. Patients were considered eligible for recruitment if they satisfied the following criteria: age above 18 years, undergoing GI endoscopy under MAC using target-controlled infusion (TCI) propofol, able to give consent, American Society of Anesthesiologists (ASA) physical status I–III. Patients were excluded if they had craniofacial abnormalities, severe cardiopulmonary diseases, a history of recent nasal or cranial bone fracture, a history of recent nasal or trans-sphenoidal surgery, nasal polyposis, a history of epistaxis, a history of coagulopathy, anticoagulants/antiplatelets therapy, a history of allergy to xylometazoline or local anesthetics, or incomplete medical history.

Patients were assigned on the day of the procedure to one of the two groups: NPA group and control group. The assignment was performed using a computer-generated random number table. The randomization results were sealed in opaque envelopes and opened sequentially, just before the induction of anesthesia. Due to the nature of the intervention, anesthesiologists and gastroenterologists could not be blinded to group allocation. However, patients were blinded, as the nasopharyngeal airway was inserted only after deep sedation had been achieved.

**Fig 1:**
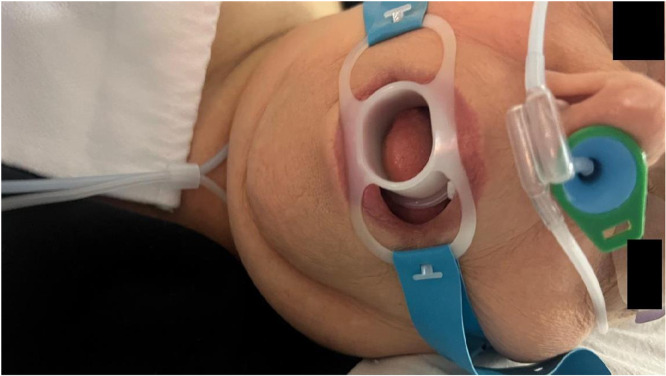
Illustration of nasopharyngeal airway (NPA) used during the procedure in combination with a nasal cannula.

Upon admission to the endoscopy unit, an intravenous line was inserted and Lactated Ringer’s infusion was started. ASA monitoring devices were attached including continuous electrocardiogram, oxygen saturation plethysmography, capnography, as well as non-invasive blood pressure monitoring every 5 minutes. In both groups, 5l/min oxygen was administered via a nasal cannula with an incorporated capnography sampling line (Flexicare® Dual Nasal Cannula). In the NPA group, the nasal cannula was positioned on top of the NPA device as shown in Fig 1. Patients were placed in the lateral decubitus position; rarely prone position was needed. All patients underwent MAC using intravenous fentanyl 1 mcg/kg, followed by propofol TCI using Marsh model. The initial plasma concentration was set at 3 μg/mL and based upon the patients’ response using the Observer’s Assessment of Alertness/Sedation (OAAS) score, up and down adjustments of 0.2 μg/mL were done [9]. Given the short duration of the endoscopic procedures. objective depth-of-anesthesia monitoring such as Bispectral Index (BIS) was not used. Instead, sedation depth was monitored using standard clinical assessment and the OAA/S score, in accordance with routine anesthesia practice.

For patients in the NPA group, a disposable NPA of appropriate size was selected by examining the distance from the tip of the patient’s nose to the tragus of the ear. Female participants were fitted with NPA devices with internal diameter (ID) 6 or 7 mm, while males had NPA devices with ID 7 or 8 mm. After ensuring that the patient was under deep sedation, the NPA was inserted. The outer surface of the airway was lubricated with lidocaine gel before insertion. Also, vasoconstriction of the mucous membranes was accomplished with xylometazoline hydrochloride (Otrivin^®^) drops in the selected nostril.

Data including age, gender, BMI, ASA score, history of pulmonary diseases and smoking were collected from the patients, in addition to the STOP-BANG screening questionnaire to identify those with obstructive sleep apnea (OSA). The STOP_BANG is validated eight-item screening tool with scores ranging from 0–8 categorized as low risk (0–2), intermediate risk (3–4), and high risk (≥5) [10]. The OSA scores were further stratified as low risk and intermediate/high risk [11]. Airway interventions including the need for chin lift, jaw thrust, bag-mask ventilation or advanced airway management (laryngeal mask airway (LMA) or tracheal intubation), and subsequent episodes of desaturation (oxygen saturation (SpO2) <90%) were recorded. Airway interventions were performed at the discretion of the anesthesiologist when apnea or clinical signs of upper airway obstruction were observed (e.g., abdominal breathing pattern and reduction or absence of EtCO_2_) for more than 10 seconds. Episodes of systolic blood pressure (SBP) <90 mmHg and heart rate (HR) <50 beats/min, development of epistaxis, signs of airway reactivity such as cough and laryngospasm, and movement or interruptions that impeded the procedure were also collected, in addition to the duration of the procedure, the total amount and dose of propofol used, and the satisfaction scores of the patient, gastroenterologist, and anesthesiologist, using a numerical scale ranging from 0 (not satisfied at all) to 10 (extremely satisfied).

The primary outcome was a composite of the occurrence of at least one airway intervention/desaturation to capture clinically relevant respiratory compromise during MAC. It included the occurrence of at least one respiratory intervention or desaturation and the individual adverse events (chin lift, jaw thrust, bag-mask ventilation, oxygen saturation <90%). The secondary outcomes were identified as NPA-related adverse events such as epistaxis, hemodynamic instability, airway reactivity, movement or interruptions, the joint effects of NPA use and OSA risk on the occurrence of at least one airway intervention and/or desaturation, as well as patient, gastroenterologist and anesthesiologist satisfaction.

### Statistical analyses

The incidence of respiratory adverse events requiring airway maneuvers during MAC varies between 14–48% [12,13]. With the assumption that 25% is the incidence of sedation-related respiratory adverse events (desaturation) [14,15], a 50% reduction was considered clinically significant. With α = 0.05 and β = 0.2, 152 patients in each group would be needed. To account for 10% drop out, we included a total of 334 patients (167 in each group).

Data were analyzed using IBM SPSS Statistics for Windows, version 29 (IBM Corp). Continuous data were checked for normality using histograms and Q-Q plots. Age, duration of procedure, and total amount of propofol were presented as means and standard deviations and analyzed using t-test. Chin lift/jaw thrust episodes as well as satisfaction scores from anesthesiologists, gastroenterologists, and patients were reported as medians with interquartile ranges and analyzed using Mann-Whitney U test. Categorical variables including BMI stratification, gender, ASA score, OSA risk groups, procedure type, history of pulmonary diseases, smoking status, incidence of airway interventions, desaturation, need for advanced airway management, incidence of movement or interruptions, SBP < 90mmHg, heart rate <50 beats/minute, epistaxis, and airway reactivity were summarized as frequencies and percentages. These were analyzed using χ2 or Fisher exact test, as appropriate. Simple logistic regression was conducted to compare the occurrence of at least one airway intervention/desaturation between the groups in the overall population, as well as within low and intermediate/high OSA risk groups. Multivariable logistic regression analysis was performed to compare the occurrence of at least one airway intervention/desaturation between the groups in the overall population and within low and intermediate/high OSA risk groups, adjusting for BMI and gender. An interaction term between groups and OSA risk was included in the logistic regression to evaluate whether the association between group and the occurrence of the primary outcome differed by OSA risk. As only five patients did not complete the intervention after randomization, the primary analysis was conducted per protocol. To evaluate the robustness of the findings, sensitivity analyses based on the intention-to-treat principle were performed by including all randomized patients in their originally assigned groups and assuming best-case and worst-case scenarios for the missing outcomes in the experimental group. In the best-case scenario, missing outcomes were assumed to have no occurrence of airway intervention or desaturation, whereas in the worst-case scenario they were assumed to have experienced the outcome. *P* value <0.05 was considered statistically significant.

## Results

**Fig 2:**
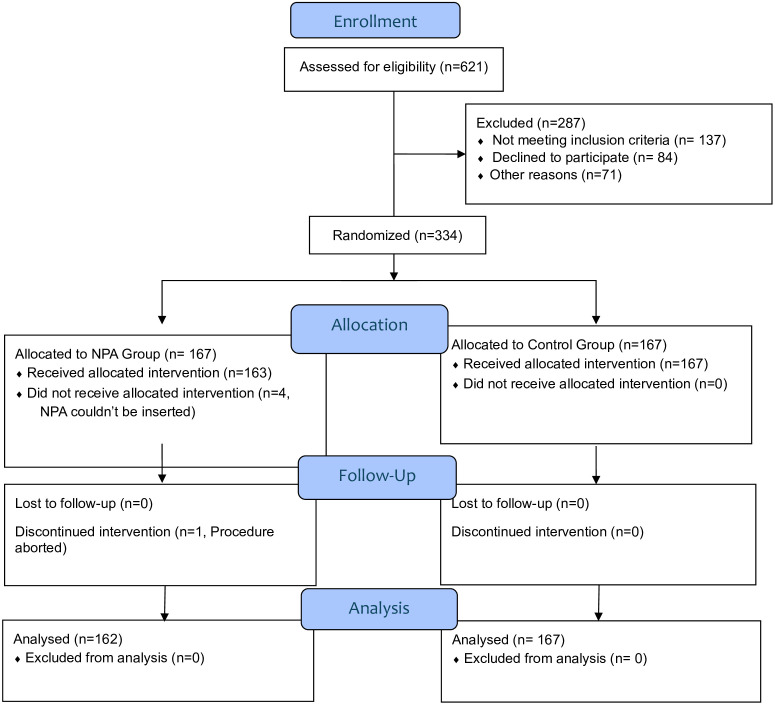
CONSORT flow diagram.

A total of 334 patients were randomized with 167 patients in each group. Four patients were excluded from the NPA group due to failure of NPA insertion, and the procedure was aborted in one additional patient. Therefore, a total of 329 patients were included in our analysis (Fig 2), with 167 patients in the control group and 162 patients in the NPA group.

The NPA group had a higher proportion of males (52.5% vs. 37.1%; Φ = 0.154) and a higher proportion of patients with high BMI (17.9% vs. 9.6%; Φ = 0.121). Other baseline characteristics were generally comparable between the groups (Table 1).

**Table 1:** Baseline characteristics of study participants.

	Control Group (n = 167)	NPA Group (n = 162)	Effect size^a^
Age (years)	55.10 ± 15.22	54.66 ± 14.39	−0.030
BMI (kg/m^2^)			0.121
Low BMI (BMI < 30 kg/m^2^)	151 (90.4)	133 (82.1)	
High BMI (BMI ≥ 30 kg/m^2^)	16 (9.6)	29 (17.9)	
Gender			0.154
Female	105 (62.9)	77 (47.5)	
Male	62 (37.1)	85 (52.5)	
ASA score			0.057
1	17 (10.2)	22 (13.6)	
2	138 (82.6)	127 (78.4)	
3	12 (7.2)	13 (8.0)	
STOP-Bang score*	2 (0-7)	2 (0-8)	−0.045 −0.025
Low OSA risk	99 (59.3)	100 (61.7)	
Intermediate/high OSA risk	68 (40.7)	62 (38.3)	
Procedure type			0.074
Endoscopy	42 (25.1)	39 (24.1)	
Colonoscopy+ Endoscopy	69 (41.3)	78 (48.1)	
Colonoscopy	56 (33.5)	45 (27.8)	
Smoking	54 (32.3)	55 (34.0)	0.017
Pulmonary diseases	11 (6.6)	13 (8.0)	0.028

Data presented as mean ± SD and n (%)

*Median (range)

^a^ Cohen’s d is reported for means, Phi (Φ) and Cramer’s V is reported for proportions, and Cliff’s delta for medians.

Table 2 depicts the procedural characteristics of the study participants. The occurrence of at least one airway intervention/desaturation < 90% was significantly lower in the NPA group compared to the control group (18.5% vs. 40.1%, *P* < 0.001), primarily driven by a reduction in the need for chin lift/jaw thrust (17.9% vs. 40.1%, P < 0.001). No significant differences were observed for bag-mask ventilation, desaturation, and chin lift / jaw thrust episodes.

**Table 2:** Procedural characteristics of the study participants.

	Control Group (n = 167)	NPA Group (n = 162)	Effect size^a^	*P*-value
Occurrence of at least one airway intervention/desaturation	67 (40.1)	30 (18.5)	−0.237	<0.001
Need for chin lift/jaw thrust	67 (40.1)	29 (17.9)	−0.244	<0.001
Need for bag-mask ventilation	6 (3.6)	1 (0.6)	−0.103	0.121
Desaturation (<90%)	12 (7.2)	14 (8.6)	0.027	0.624
Chin lift/jaw thrust episodes*	3 (1-12)	2 (1-8)	−0.198	0.057
SBP < 90 mmHg	16 (9.6)	10 (6.2)	−0.063	0.252
Heart rate <50 beats/min	7 (4.2)	1 (0.6)	−0.116	0.067
Epistaxis	0 (0.0)	5 (3.1)	0.126	0.028
Cough	7 (4.2)	3 (1.9)	−0.068	0.337
Laryngospasm	1 (0.6)	0 (0.0)	−0.054	1.000
Interruptions during the case	4 (2.4)	1 (0.6)	−0.073	0.371
Movement impeding the procedure	8 (4.8)	4 (2.5)	−0.062	0.262
Duration of procedure (min)	35.0 ± 17.3	34.4 ± 13.4	−0.043	0.698
Total amount of propofol (mg)	293.7 ± 150.3	315.4 ± 124.6	0.157	0.155
Total dose of propofol (mg/kg/hr)	7.95 ± 4.57	8.11 ± 3.74	0.04	0.716
Anesthesiologists’ satisfaction (0–10) *	9 (4-10)	10 (5-10)	0.452	<0.001
Gastroenterologists’ satisfaction (0–10) *	9 (5-10)	10 (4-10)	0.281	<0.001
Patients’ satisfaction (0–10) *	10 (7-10)	10 (7-10)	0.005	0.847

Data presented as mean ± SD and n (%)

*Median (range)

^a^ Cohen’s d is reported for means, Phi (Φ) and Cramer’s V is reported for proportions, and Cliffs’ delta is reported for ordinal data.

Among secondary outcomes, epistaxis occurred in 5 patients (3.1%) in the NPA group, whereas no cases were reported in the control group (P = 0.028).

Moreover, anesthesiologists’ and proceduralists’ satisfaction scores were significantly higher in the NPA group compared to the control group (10 (5–10) vs. 9 (4–10), P < 0.001; 10 (4–10) vs. 9 (5–10), P < 0.001, respectively). Patient satisfaction scores did not differ significantly between groups (P = 0.847).

Table 3 shows the joint effects of NPA use and OSA risk, on the occurrence of at least one airway intervention/desaturation. After adjusting for BMI and gender, the occurrence of at least one airway intervention/desaturation was significantly lower in the NPA group compared to the control group (adjusted OR=0.30, 95% CI [0.18, 0.51]). The interaction between the control group and OSA classification was significant (*P* < 0.0001), and the efficacy of NPA in reducing the occurrence of the primary outcome was significantly stronger in low OSA risk group (adjusted OR=0.23, 95% CI [0.11, 0.49]) compared to the intermediate/high OSA risk group (adjusted OR=0.45, 95% CI [0.21, 0.95]), while adjusting for BMI and gender.

**Table 3:** Joint effects of NPA use and OSA risk on the occurrence of at least one airway intervention/desaturation.

	Occurrence of at least one airway intervention/desaturation		OR^†^	95% CI	OR^‡^	95% CI
	Yes	No				
*Based on OSA risk*						
Overall			0.34	[0.21, 0.56]	0.30	[0.18, 0.51]
Control	67 (40.1)	100 (59.9)				
Experimental	30 (18.5)	132 (81.5)				
Low OSA risk			0.23	[0.11, 0.48]	0.23	[0.11, 0.49]
Control	35 (35.4)	64 (64.6)				
Experimental	11 (11.0)	89 (89.0)				
Intermediate/ high OSA risk			0.50	[0.24, 1.02]	0.45	[0.21, 0.95]
Control	32 (47.1)	36 (52.9)				
Experimental	19 (30.6)	43 (69.4)				

Data presented as n (%)

^†^ Crude OR

^‡^ OR adjusted for BMI and gender

### Sensitivity analysis

Sensitivity analyses assuming best-case and worst-case outcomes for the five patients who did not complete the intervention yielded results consistent with the primary analysis (best-case adjusted OR=0.30, 95% CI [0.17–0.49]; worst-case adjusted OR=0.35, 95% CI [0.21–0.57]), indicating that the missing data did not affect the study conclusions.

## Discussion

While desaturation alone did not differ significantly between groups, our study showed that the routine use of NPA during GI endoscopy reduces the occurrence of airway interventions in patients undergoing GI endoscopy, while increasing anesthesiologist and proceduralist satisfaction.

One of the most common complications associated with sedation during endoscopy is respiratory compromise [16]. This can be explained in part by the use of sedation which can cause hypopnea [17] and in another part by the nature of GI endoscopy which may compromise and block the airway [6]. Supraglottic airway devices have been recommended by the American Heart Association as a first choice in emergent resuscitation [18] but have not been used routinely in the setting of GI endoscopy. Due to the possibility of using NPAs in semiconscious and unconscious patients, we aimed to identify the value of the routine use of NPA in the context of MAC for GI endoscopy.

A study by Muller et al showed positive outcomes with a decrease in desaturation episodes from 13.5% in the control group to 1.9% in the NPA group [1]. However, this study does not mention the use of airway maneuvers prior to occurrence of desaturation, which might be due to sedation being administered by non-anesthesiologist physicians. Our study did not rely solely on the occurrence of desaturation events but also captured the need for airway interventions, which are typically performed early in response to developing airway obstruction. By incorporating these interventions as outcomes, the study provides a more sensitive assessment of NPA efficacy than the evaluation of desaturation alone. The positive outcomes of our study build on the existing literature to further reinforce the benefits of routine NPA use in GI endoscopy procedures.

We conducted a subgroup analysis to evaluate the effectiveness of NPA use in patients at higher risk of perioperative adverse airway events, particularly those with intermediate/high OSA risk. Our results showed that NPA reduced the need for airway maneuvers in both low (adjusted OR=0.21, 95%CI [0.09, 0.46]) and intermediate/high OSA (adjusted OR=0.36, 95%CI [0.17, 0.80]) risk groups. However, the reduction was stronger in the low OSA risk group. This could be explained by the higher baseline risk of respiratory adverse events in patients with OSA [19,20].

While NPA use reduced the primary outcome, the residual incidence in the intermediate/high OSA risk group remained higher than that achieved in the low OSA risk group (30.6% vs. 11%). In fact, during sleep, patients with sleep apnea suffer from recurrent episodes of partial or complete obstruction of the upper airway that occur when the negative pressure of inspiratory muscles exceeds the dilator muscle activity of the upper airway. The administration of sedatives, analgesics and anesthetics in OSA patients aggravates obstruction of the pharynx. In OSA patients under anesthesia, depression of the upper airway muscles is greater than that of the diaphragm. Thus, breathing efforts continue while upper airway muscle activity is significantly reduced, which predisposes them to upper airway collapse during inspiration. Furthermore, anesthetics impair the arousal response, a defense mechanism that protects against sleep apnea by helping overcome the airway obstruction [20]. The use of NPA in this patient population can help alleviate the airway obstruction caused by upper airway collapse. However, it does not address the impaired arousal response, which contributes to central apnea. Consequently, and following the most recent guidelines, in patients with any predictors of difficult airway including OSA, high flow nasal cannula should be considered as a first line oxygenation technique, as it increases functional residual capacity and, thus, the total volume of oxygen available during apnea [21,22].

Our study demonstrated that the routine use of NPA in GI endoscopy is safe. Epistaxis occurred in 3.1% of the cases. However, these episodes were mild, self-limiting, required no intervention, resolved within three minutes, and didn’t interfere with endoscopic visualization or the completion of the procedure. Additionally, NPA insertion was unsuccessful in 2.3% of the cases. In the study by Xiao et al., no cases of epistaxis were reported with NPA use, but they observed a similar 2.3% failure rate of NPA insertion and highlighted the importance of avoiding forceful insertion to prevent complications [7]. In our study, we carefully selected eligible patients by excluding those with coagulopathy, history of epistaxis, anticoagulant use, or craniofacial abnormalities or disease. Proper patient selection, along with gentle NPA insertion, is crucial for minimizing complications and ensuring safe use.

Our study showed a significant increase in gastroenterologist and anesthesiologist satisfaction with the use of NPA during GI endoscopy. This can be attributed to the decreased need for airway maneuvers, making the work of the anesthesiologist easier and decreasing distractions for the proceduralist. Furthermore, although patients’ satisfaction was assessed using a numerical scale from 0–10 and did not specifically evaluate for nasal discomfort, the potential for nasal soreness associated with NPA use did not appear to influence overall patient satisfaction, as no difference in satisfaction scores was observed between the control and NPA groups.

Additionally, the NPA is a relatively non costly device and does not necessitate an increase in drug dosing. When used appropriately, it can be inserted and removed quickly and thus does not increase procedure time.

This study does have some limitations that need to be addressed. First, our study is a single-blinded RCT. The nature of this study does not allow for a double blinded RCT. As such, the lack of blinding of the anesthesia provider assessing outcomes may introduce potential performance and detection bias. Although a standardized protocol for the intervention (chin lift/jaw thrust) was implemented, the possibility of subjective clinical judgment (e.g., defining apnea >10 seconds) cannot be excluded. A second limitation is the significantly higher proportion of males and a higher BMI in the NPA group, which are risk factors for airway obstruction during sedation [23,24]. Despite this imbalance, NPA use significantly decreased the need for airway interventions. A more balanced distribution of these variables between groups might have revealed an even stronger association. Although BMI and gender were adjusted for in the multivariable analysis, this imbalance may have impacted the magnitude of the reduction. Moreover, we cannot exclude the possibility of residual confounding from other factors such as ASA status and procedure type. Third, most participants had relatively low BMI and ASA I–II status, which may limit the generalizability of our findings to higher-risk populations, including patients with severe obesity, advanced comorbidities, or those undergoing more complex endoscopic procedures. Furthermore, a limitation of this study is the potential for provider-dependent variability in the performance of airway maneuvers. Although airway interventions were performed after 10 seconds of apnea or airway obstruction, a formalized protocol specifying the exact criteria for initiating them was not implemented and their initiation involved some degree of subjective clinical judgment. Nevertheless, participating centers adhered to standard anesthesia monitoring and airway management practices consistent with their institutional protocols and all clinicians involved in patient care were certified anesthesiologists with standard training in airway management. Finally, in contrast to the use of NPA which only relieves airway obstruction, airway maneuvers could reverse both airway obstruction and central apnea, and our study was not designed to differentiate between the two. Exploring the root cause for airway interventions may help refine the needed interventions and lead to more precise conclusions.

### Conclusion

In conclusion, our study results demonstrate that NPA use during GI endoscopy significantly reduces the incidence of airway interventions, particularly in patients at low risk for OSA. Our findings also highlight an improvement in satisfaction levels among anesthesiologists and gastroenterologists when utilizing NPAs, suggesting that this intervention not only enhances patient safety but also promotes a more efficient and satisfactory procedural experience.

Although NPA use showed a favorable safety profile, a small proportion of patients experienced mild, self-limiting epistaxis, highlighting the importance of careful patient selection and gentle insertion techniques. Considering its effectiveness in reducing incidence of airway interventions, coupled with the cost-effectiveness and ease of NPA insertion, NPA may represent a useful adjunct for airway management during GI endoscopy under MAC. Future studies may provide further insights into the root cause of airway events (central vs. obstructive) and define the exact role of NPA in the context of various baseline risk factors such as OSA.

## Abbreviations

ASA: American Society of Anesthesiologists
BMI: Body mass index
EUS: Endoscopic Ultrasound
GI: Gastrointestinal
HR: Heart rate
ID: Internal Diameter
LMA: Laryngeal mask airway
MAC: Monitored Anesthesia Care
NPA: Nasopharyngeal Airway
OAAS: Observer’s Assessment of Alertness/Sedation
OSA: Obstructive Sleep Apnea
RCT: Randomized controlled trial
SBP: Systolic blood pressure
SpO2: Oxygen saturation
TCI: Target controlled infusion.
